# The P2X7 Receptor Regulates IL-1β Secretion in the Human Retina

**DOI:** 10.3390/ijms262110345

**Published:** 2025-10-23

**Authors:** Nuwan Niyadurupola, Peter Sidaway, David C. Broadway, Julie Sanderson

**Affiliations:** 1 Department of Ophthalmology, Norfolk and Norwich University Hospitals NHS Foundation Trust, Colney Lane, Norwich NR4 7UY, UK; nuwan.niya@doctors.org.uk (N.N.);; 2 School of Chemistry, Pharmacy and Pharmacology, University of East Anglia, Norwich NR4 7TJ, UK

**Keywords:** P2X7, interleukin1-beta, neuroinflammation, human organotypic retinal cultures, retina, glaucoma

## Abstract

The P2X7 receptor has been associated with the neurodegeneration of retinal ganglion cells (RGCs), which is central to the loss of vision in glaucoma. Furthermore, the activation of P2X7 has been shown to cause the death of RGCs, including in the human retina. Human organotypic retinal cultures (HORCs) were used to investigate the potential indirect mechanisms of RGC death. Of the 27 cytokine/growth factors assayed, the stimulation of P2X7 using BzATP (100 µM; 36 h) significantly increased the secretion of IL-1β and IL-10. IL-1β was selected for further investigation. BzATP (100 µM) caused an increase in the expression and release of IL-1β in a time-dependent manner; this increase was inhibited through a co-incubation with BBG (1 µM). Exogenous IL-1β alone (10 ng/mL) did not cause a loss of RGCs. However, IL-1β inhibited the loss of RGCs caused by BzATP, and this neuroprotection was prevented by the Interleukin-1 receptor-1 antagonist (IL-1ra) (100 ng/mL). The IL1 receptor IL-1R1 was localised to the inner retina close to the RGCs, although not predominantly co-localised with RGC bodies. The results suggest that the P2X7-mediated death of RGCs is not IL-1β mediated. Furthermore, IL-1β may be upregulated as part of a response to mitigate P2X7-mediated damage to the retina. Our research is the first to indicate the P2X7-mediated regulation of IL-1β in the human retina and supports the role of the ATP/P2X7/IL-1β axis in RGC survival and possible glaucomatous RGC degeneration.

## 1. Introduction

The P2X7 receptor is a ligand-gated non-selective cation channel activated by extracellular adenosine triphosphate (ATP) [1]. In one of its key roles, P2X7 is expressed in immune cells, including macrophages and microglia, contributing to the inflammatory response [2,3,4] where the stimulation of the receptor leads to IL-1β secretion [5,6,7,8] via the activation of the NLRP3 inflammasome [9]. P2X7 activation has been implicated in neuronal cell death in a number of in vivo and in vitro central nervous system (CNS) model systems [4,10,11], including in the retina [12,13,14,15,16,17,18,19]. With mechanisms comparable to glutamate excitotoxicity via NMDA receptors, the direct stimulation of P2X7 in neuronal cell membranes can cause a neuronal Ca^2+^ overload and consequent cell death [11,12]. In addition, P2X7 activation contributes to neuroinflammatory pathways and downstream neurodegeneration [3,4]. P2X7-mediated mechanisms are implicated in neurodegenerative diseases including Alzheimer’s disease, Parkinson’s disease, multiple sclerosis and glaucoma, and it has been suggested that P2X7 is a target of interest in the treatment of these diseases [4,20,21]. Of dual interest is the prevention of ATP-mediated “excitotoxic” cell death, together with the mitigation of neuroinflammatory pathways. However, the mechanisms are complex, and a fuller understanding is required. For example, although the vast majority of previous publications focus on the release of pro-inflammatory cytokines, there are also reports of P2X7 receptors contributing to the resolution of inflammation [22,23,24]. In addition, the characterisation of P2X7 across different species reveals variations in the structure, function and pharmacology of the receptor [1]. Findings in animal models, therefore, may not be replicated in humans and, more importantly, may not therefore translate to human disease. Therefore, experimental data using human tissue models are valuable for validating and informing the discussion of the role of P2X7 in the neurodegenerative processes of human disease.

The retina constitutes part of the CNS. Photoreceptors transduce light, and retinal neurons perform the initial processing before retinal ganglion cell (RGC) axons transmit information to the brain via the optic nerve [25]. The retinal neurons are supported by microglia and astrocytes as well as Müller cells, which are retinal-specific glial cells [26]. Glaucoma is a neurodegenerative disease of the retina in which there is a progressive loss of RGCs [27]. Since the axons of RGCs form the optic nerve, the death of these neurons leads to visual field loss and eventual blindness. An elevated intraocular pressure (IOP) is the major risk factor associated with glaucoma [28]. However, glaucoma can occur without the elevation of IOP and does not always respond, or fully respond, to a reduction in IOP, providing evidence for IOP-independent risk factors. The stimulation of retinal P2X7 has been associated with RGC death in a number of in vivo models of glaucoma [14,15,16,17,18,19,29,30,31]. Furthermore, increased levels of extracellular ATP have been demonstrated in the posterior segment of the eye in models of glaucoma [14,32], including in non-human primates [32]. Exposure to P2X7 antagonists [15,17,19,29,30,31] and P2X7-/- knockout have been reported to ameliorate RGC death [16]. The activation of microglia is a consistently observed feature in models of glaucomatous neurodegeneration [33,34], and activated microglia have been observed in the optic nerve head of human glaucomatous eyes [35]. Moreover, in models using an increased IOP to induce glaucoma, this activation has been found to be P2X7-dependent [15,16,17,31], and a direct stimulation of P2X7 in the in vivo and ex vivo retina and in primary cultures of microglial cells have been shown to cause morphological change and the upregulation of markers linked to microglial activation [16]. P2X7 is associated with the secretion of the pro-inflammatory cytokine, interleukin-1β (IL-1β) [5,6,7], and IL-1β has been reported to be associated with glaucomatous RGC death [36,37,38,39,40,41]. Furthermore, the upregulation of retinal IL-1β observed in rodent models with increased IOP has been shown to be P2X7-dependent [17,42]. By interfering with IL-1β signalling using the IL-1 receptor-1 antagonist (IL-1ra), anti-IL-1β antibodies or siRNA reduced RGC death in rodent eyes, indicating that IL-1β was detrimental to RGC survival [36,37,38,40]. However, the converse has also been found, with IL-1β being shown to be protective for RGCs in in vivo and ex vivo models using rodent eyes [37,43,44,45].

Using human organotypic retinal cultures (HORCs) [46,47], we have previously found that P2X7 stimulation causes a loss of neuronal cells from the retinal ganglion cell layer [13]. The hypothesis investigated in the present study was whether the P2X7-induced RGC loss was mediated indirectly via cytokine release as a result of P2X7 activation. With experimental systems centred around rodent models yielding significant data on the role of P2X7 in neuroinflammatory processes, it was novel to determine whether equivalent pathways exist in the human retina that could contribute to the pathophysiological processes mediating the loss of vision in glaucoma. Our findings indicated, for the first time, that P2X7 activation caused IL-1β release in the human retina. Intriguingly, the results indicated that the release of IL-1β was not responsible for P2X7-mediated RGC degeneration in the human retina, but, in fact, had neuroprotective actions. The findings confirmed the presence of the ATP/P2X7/IL-1β axis in the human retina and, more widely, in human CNS tissue.

## 2. Results

### 2.1. The Profile of Cytokine and Growth Factor Secretion from BzATP-Stimulated HORCs

The investigation of the secretion of 27 cytokines and growth factors revealed that only IL-1β and IL-10 protein levels were significantly elevated following the incubation of HORCs with the P2X7 agonist, BzATP (100 µm), for 36h compared with the controls (*p* < 0.05; *n* = 4) (Table 1). IL-1β protein levels were 3-fold higher in the culture medium of HORCs exposed to BzATP compared with the controls (*p* < 0.05; *n* = 4). The levels of IL-1ra secreted into the medium were unchanged.

### 2.2. BzATP Induces IL-1β mRNA Expression and IL-1β Protein Release

**Figure 1:**
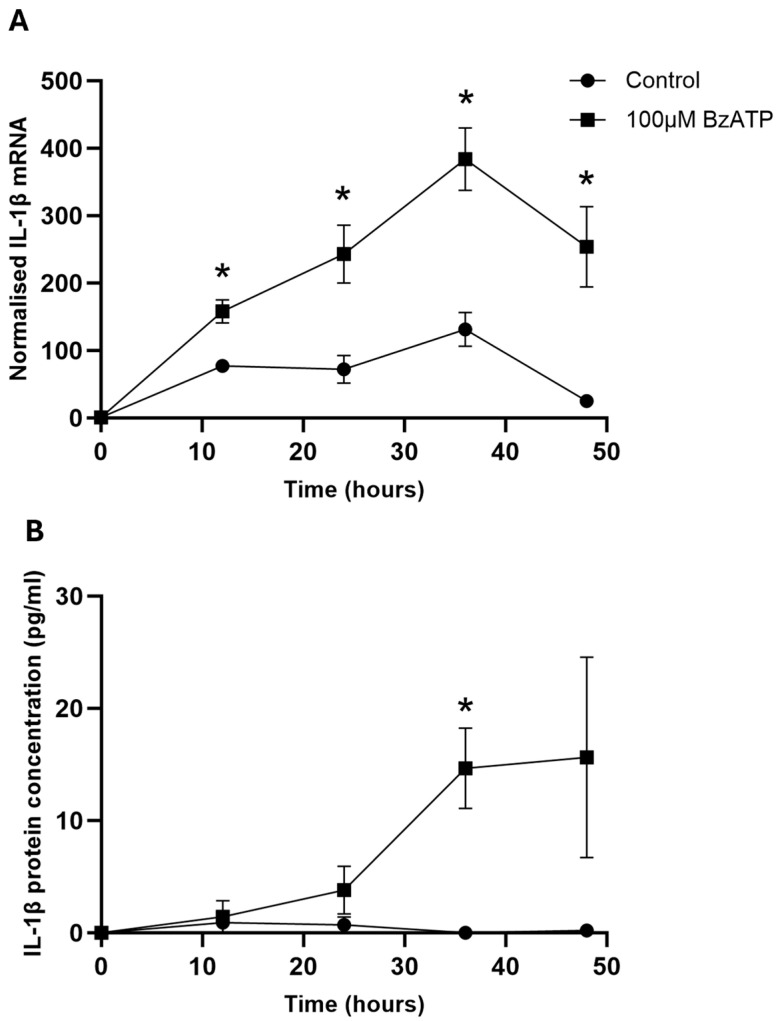
(**A**) Normalised IL-1β mRNA expression of control and 100 μM BzATP-stimulated HORCs at 0 h (*n* = 39), 12 h (*n* = 24), 24 h (*n* = 27), 36 h (*n* = 16) and 48 h (*n* = 4) in culture. IL-1β mRNA expression was normalised relative to the expression of TOP1 and CYC1 mRNA. (**B**) IL-1β protein concentration in the medium from control and 100 μM BzATP-stimulated HORCs measured at 0, 12, 24, 36 and 48 h in culture (*n* = 4). Data are presented as mean ± SEM; * *p* < 0.05; Student’s *t*-test.

The incubation of HORCs with 100 µM BzATP caused a significant increase in IL-1β mRNA expression at all timepoints measured (12, 24, 36 and 48 h) compared with the control HORCs (Figure 1A). The greatest level of IL-1β mRNA expression was found after 36 h of incubation with 100 µM BzATP, where an approximate 3-fold increase in expression was seen compared with the control levels.

Over the same time course, there was very little IL-1β protein secretion into the culture medium by the control HORCs (Figure 1B). In contrast, HORCs stimulated with 100 µM BzATP showed increased IL-1β protein levels in the culture medium, with a significant increase at 36 h compared with the controls (*p* < 0.05) (Figure 1B).

### 2.3. Involvement of P2X7 in IL-1β mRNA Expression and IL-1β Protein Release

**Figure 2:**
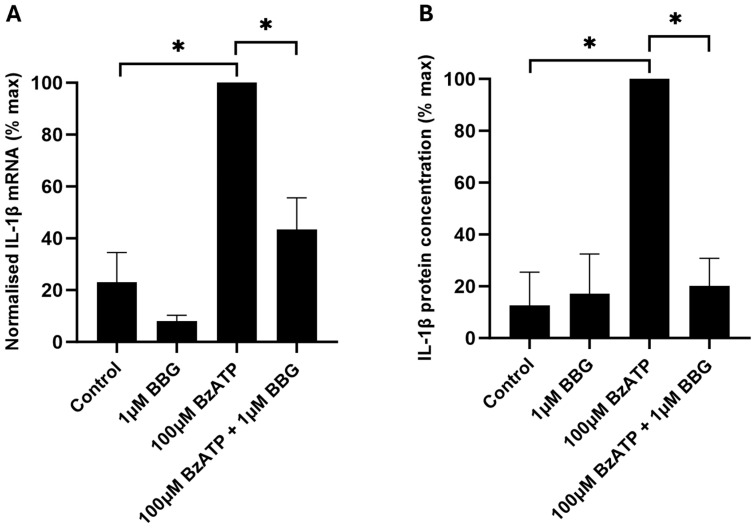
(**A**) IL-1β mRNA expression (% max) and (**B**) IL-1β protein concentration (% max) of control, 1 μM BBG-treated, 100 μM BzATP-treated and 1 μM BBG + 100 μM BzATP-treated HORCs at 36 h. IL-1β mRNA expression was normalised relative to the expression of TOP1 and CYC1 mRNA. Data are presented as mean ± SEM; *n* = 4; * *p* < 0.05; ANOVA with post hoc Tukey comparison.

To investigate whether the BzATP-induced increase in IL-1β mRNA expression and secretion was mediated by P2X7, BBG was utilised. BBG has been reported to be a non-competitive antagonist of P2X7 with a selectivity over other P2X receptors [48], and we have shown previously that it inhibits BzATP-induced RGC death in the human retina [13]. BBG (1 μM) significantly inhibited the 100 µM BzATP-induced increase in IL-1β mRNA expression of HORCs at 36 h (*p* < 0.05) (Figure 2A). Similarly, 1 µM BBG completely inhibited the BzATP-induced secretion of IL-1β protein by HORCs at 36 h (*p* < 0.05) (Figure 2B).

### 2.4. IL-1β Protects RGCs from BzATP-Induced Cell Death

**Figure 3:**
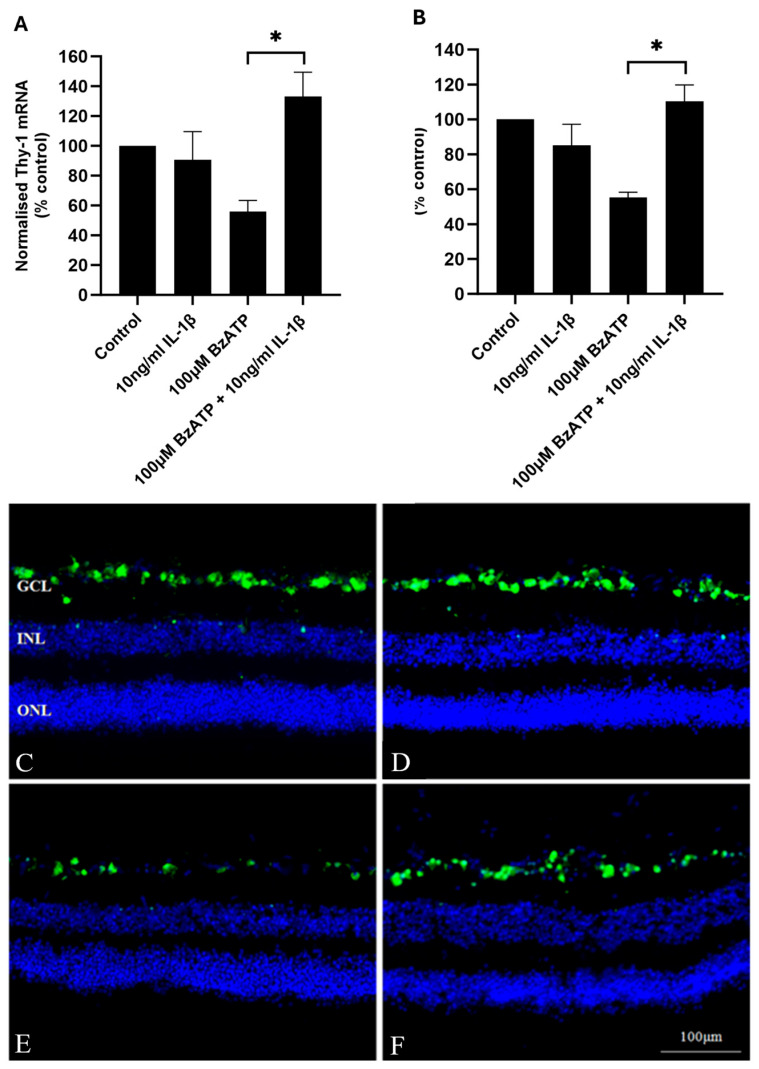
(**A**) Normalised *THY-1* mRNA (% control; *n* = 8) and (**B**) NeuN-labelled neuronal cell counts in 200 μm (% control; *n* = 3) of control, 10 ng/mL IL-1β-treated, 100 μM BzATP-treated and 100 μM BzATP + 10 ng/mL IL-1β-treated HORCs at 24 h. *ThY-1* mRNA expression was normalised relative to the expression of *TOP1* and *CYC1* mRNA. Data are presented as mean ± SEM; * *p* < 0.05; ANOVA with post hoc Tukey comparison. (**C**–**F**) Representative immunofluorescence photomicrographs of (**C**) control, (**D**) 10 ng/mL IL-1β-treated, (**E**) 100 μM BzATP-treated and (**F**) 100 μM BzATP + 10 ng/mL IL-1β-treated HORCs at 24 h stained with NeuN (green) and the nuclear stain DAPI (blue). GCL, ganglion cell layer; INL, inner nuclear layer; ONL, outer nuclear layer.

In order to assess whether IL-1β had any effect on RGC death, exogenous IL-1β (10 ng/mL) was co-incubated with BzATP, and the number of RGCs was assessed by quantifying the mRNA expression of the RGC marker *THY-1*, as well as counting the number of NeuN-positive RGCs. These techniques have been shown previously to accurately reflect RGC numbers in HORCs [46,47]. The incubation of HORCs with 10 ng/mL IL-1β alone for 24 h did not affect *THY-1* mRNA or the number of NeuN-positive neuronal cells in the RGC layer compared with the controls at 24 h (Figure 3). However, 10 ng/mL IL-1β completely inhibited the BzATP-induced loss of *THY-1* mRNA from HORCs at 24 h (*p* < 0.05) (Figure 3A). Similarly, 10 ng/mL IL-1β completely inhibited the loss of NeuN-positive neuronal cells from the RGC layer of HORCs at the same timepoint (*p* < 0.05) (Figure 3B–F).

**Figure 4:**
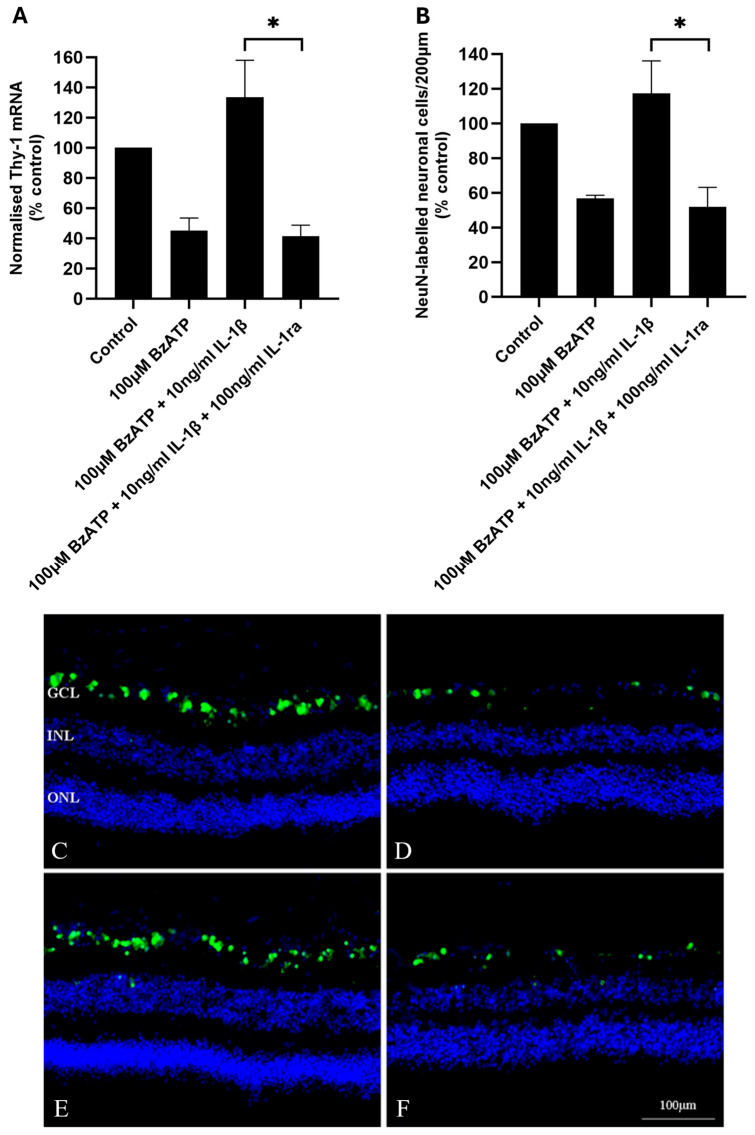
(**A**) Normalised *THY-1* mRNA (% control; *n* = 4) and (**B**) NeuN-labelled neuronal cell counts in 200 μm (% control; *n* = 3) of control, 100 μM BzATP-treated, 100 μM BzATP + 10 ng/mL IL-1β-treated and 100 μM BzATP + 10 ng/mL IL-1β + 100 ng/mL IL-1ra-treated HORCs at 24 h. *THY-1* mRNA expression was normalised relative to the expression of *TOP1* and *CYC1* mRNA. Data are presented as mean ± SEM; * *p* < 0.05; ANOVA with post hoc Tukey comparison. (**C**–**F**) Representative immunofluorescence photomicrographs of (**C**) control, (**D**) 100 μM BzATP-treated, (**E**) 100 μM BzATP + 10 ng/mL IL-1β-treated and (**F**) 100 μM BzATP + 10 ng/mL IL-1β + 100 ng/mL IL-1ra-treated HORCs at 24 h stained with NeuN (green) and the nuclear stain DAPI (blue). GCL, ganglion cell layer; INL, inner nuclear layer; ONL, outer nuclear layer.

To determine whether the protective effect of IL-1β on P2X7-mediated RGC death in HORCs was due to the activation of the IL-1 receptor IL-1R1, HORCs were pre-incubated with IL-1ra (100 ng/mL; 1 h) prior to the addition of BzATP (100 µM) in the presence of IL-1ra. The antagonist completely inhibited the protective effect of IL-1β (*p* < 0.05), with similar levels of *THY-1* mRNA and NeuN-positive neuronal cells in the RGC layer in comparison with HORCs exposed to 100 µM BzATP alone (Figure 4).

### 2.5. IL-R1-Immunoreactivity Was Found Principally in the Retinal Nerve Fibre and RGC Layers of Human Retina

**Figure 5:**
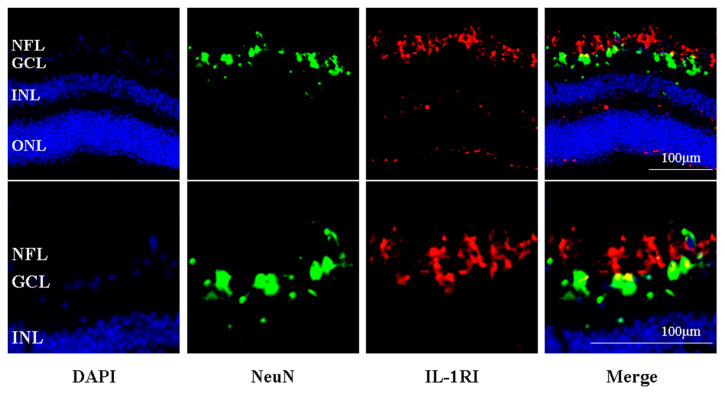
Immunofluorescence photomicrograph of a HORC at 0 h (above), with an enlarged image of the retinal ganglion cell layer (below) stained for IL-1RI (red), NeuN (green) and the nuclear stain DAPI (blue). GCL, ganglion cell layer; INL, inner nuclear layer; ONL, outer nuclear layer. The degree of co-localization of IL-1R1 and NeuN was 2.1%. Pearson’s coefficient, *r* = 0.43.

The IL-1R1 immunoreactivity was localised principally in the retinal nerve fibre and RGC layers of human retina (Figure 5). The majority of IL-1R1 immunoreactivity did not co-localise with the NeuN-positive cell bodies of the RGC layer and was mainly on the inner side of the RGC layer. To a lesser extent, IL-1R1 immunoreactivity was also found in the outer plexiform layer of the retina and in the outer aspect of the outer nuclear layer of the human retina.

## 3. Discussion

Neuroinflammation is recognised as a fundamental process in retinal pathologies, including glaucoma. The present study aimed to investigate P2X7-dependent cytokine secretion in the human retina and whether this contributed to P2X7-mediated cell death. The novel finding was that, in the human retina, the activation of P2X7 leads to an IL-1β release. The activation of P2X7 has been previously shown to be detrimental to cells of the RGC layer in rodent and human retina [13,14,15,16,17,18,19]. Of the 27 cytokines and growth factors assayed, the incubation of HORCs with the P2X7 agonist BzATP resulted in a significant increase in the secretion of IL-1β and IL-10. In studies of the retina, IL-10 has been shown to have a neuroprotective effect [49,50], whereas IL-1β is reported to be principally neurotoxic [17,36,37,38,40,41]. Interleukin-1β levels have also been shown to increase in the aqueous humour of patients with glaucoma [51], and single-nucleotide polymorphisms in the IL-1β gene have been associated with a risk of glaucoma [52]. Therefore, IL-1β was investigated further as a potential mediator of the neurotoxic effects of P2X7 receptor activation in the human retina.

IL-1β mRNA and protein concentrations were significantly elevated following the stimulation of HORCs with BzATP, and this effect was inhibited by the P2X7 antagonist, BBG. The results indicated that the activation of P2X7 in HORCs increased IL-1β production at the transcriptional level and also increased IL-1β protein secretion. The IL-1β mRNA and secreted protein concentrations were maximal after a 36 h exposure of HORCs to BzATP. Interleukin-1β expression under control conditions remained low, indicating that expression, as well as the release of IL-1β, was P2X7-dependent. P2X7 activation is a recognised trigger for IL-1β release, including from primary immune and non-immune cells of human origin [5,53,54,55,56,57]. There is less documentation that P2X7 activation also increases the expression of IL-1β, although this effect has been reported, for example, in microglia [58,59] and astrocytes [42]. Furthermore, a recent publication demonstrated a P2X7-mediated increase in IL-1β expression and release in a murine organotypic retinal explant model [59]. The research presented here further supports the role of extracellular ATP and P2X7 activation in both the production and release of IL-1β.

Since HORCs retain all the cells of the human retina, the candidate cell types for the production and release of IL-1β are multiple. The release of IL-1β following P2X7 activation has been shown in rat retinal microglia [59,60] and also in ONH astrocytes [42]. In vivo, retinal microglia and astrocytes, as well as Müller cells, RGCs, vascular endothelial cells and recruited neutrophils, have also been shown to upregulate IL-1β in response to retinal stressors [31,40,59,61]. The NLRP3 inflammasome has been identified in retinal cells, including microglia, Müller cells, astrocytes and RGCs [31,42,62]. Any of these cell types, therefore, could be contributing to IL-1β production and release in our organotypic system. Our presented research, to our knowledge, is the first to demonstrate that IL-1β is upregulated and released in the human retina when P2X7 receptors are activated. It appears, therefore, that P2X7 is able to invoke neuroinflammatory mechanisms in the human retina. Our findings add to the results from animal model studies that have demonstrated P2X7 activation to be central to neuroinflammation and neurodegeneration in the retina [16,17,31].

In order to investigate the effect of IL-1β on human RGCs, experiments in HORCs applying exogenous IL-1β were carried out. It was anticipated that IL-1β would cause RGC death; however, results were to the contrary: IL-1β protected RGCs from P2X7-induced cell death. The protective effect of IL-1β is intriguing and suggests that, whilst P2X7 stimulation is neurotoxic, pathways may also be activated that aim to mitigate against neurodegenerative activity. Although most of the literature suggests a neurotoxic role for IL-1β, it has been found to have a neuroprotective effect on RGCs in numerous retinal studies. For example, in vivo neuroprotection following optic nerve transection has been demonstrated, with IL-1β promoting the survival of axotomised rat RGCs [43]. In addition, in both murine in vivo [45] and murine retinal explant [44] models, IL-1β protected RGCs from glutamate excitotoxicity [44]. Interestingly, Kido and co-workers [37] found IL-1β to be both neurotoxic and neuroprotective to the rat retina in vivo in different experiments. These dual neurotoxic and neuroprotective effects of IL-1β have also been shown with different experimental conditions in rat organotypic hippocampal slice cultures [63]. A considerable interest exists in the role of IL-1β in glaucoma, including a consideration of targeting IL-1β production as a therapeutic strategy [64]. In the context of therapeutic avenues, it is important to recognise the complexity of the role of IL-1β. When IL-1β is found to be upregulated in models of retinal pathology, or, indeed, in reports of increased levels in human retinal pathophysiology, the potential neuroprotective actions should be considered.

It is important to address the question of why the upregulation of IL-1β that was observed was not able to mitigate RGC loss in our experimental system: IL-1β secretion was increased by P2X7 activation and exogenous IL-1β was shown to be neuroprotective, yet P2X7 activation was shown to result in RGC death. The explanation likely lies with the timings observed for the upregulation of IL-1β following P2X7 activation. On application of BzATP, there was a significant increase in the expression of IL-1β mRNA from 12 to 48 h. However, this was not translated to an increase in released IL-1β protein concentration until the 36 h timepoint. The experiments investigating RGC loss with BzATP were conducted over 24 h and showed that RGC numbers decreased within this timeframe, and therefore there was not the possibility for the “endogenous” release of IL-1β to show any protective effects. It should also be added that the concentration of IL-β reached in the experiment is lower than was added exogenously, which could also be significant. However, local tissue concentrations of the secreted IL-1β are likely to be considerably higher in the limited extracellular space within the retina compared to that measured in the large volume of medium in which the HORCs were cultured under the experimental conditions.

The protective effects of IL-1β on P2X7-related neurotoxicity observed here in human RGCs was dependent on the activation of the IL-1R1, since inhibition of the IL-1R1 with IL-1ra completely inhibited these effects. The IL-1R1 was principally located in the RGC and nerve fibre layers of the human retina, with some IL-1R1-immunoreactivity in the outer plexiform layer and the outer aspect of the outer nuclear layer. Previously, the IL-1R1 has been localised to the RGCs of the neonatal rat retina [41], and some co-localization of IL-1R1 with Neu-N labelled neurones of the RGC layer of the human retina was observed in the present study. A direct neuroprotective effect of IL-1β on a subset of RGCs is therefore a possibility. However, the majority of IL-1R1-immunoreactivity was not localised in the cell bodies of RGCs, indicating that the expression is more likely in non-neuronal cells in this region. Interleukin-1R1 expression has been found in astrocytes and Müller cells of the inner retina of mice [45], which is consistent with the pattern of expression seen in the present study of the human retina. Furthermore, stimulation of the IL-1R1 with IL-1β has been shown to protect RGCs from glutamate excitotoxicity [44,45], again consistent with the data presented here demonstrating IL1β to be neuroprotective via the activation of the IL-1R1. Both Namekata et al. [44] and Todd et al. [45] found that neuroprotection was via indirect glial-mediated mechanisms, implicating Müller cells and astrocytes in the initial IL-1β response. It is also interesting that P2X7 stimulation has been shown to be involved in the release of IL-3 from RGCs, with IL-3 having neuroprotective actions [65]. P2X7-dependent IL-6 release has also been demonstrated in retinal cells [66,67]. As with IL-1β, IL-6 is another cytokine with context-dependent effects on neuronal survival [68], further demonstrating the complex role of the P2X7 receptor in neuronal survival.

Both the time course of BzATP-induced IL-1β secretion and the neuroprotective effects of “exogenous” IL-1β in the present study suggest that the increase in IL-1β release is not the mechanism of P2X7-mediated RGC death observed in the human retina. The mechanisms responsible for P2X7-mediated RGC death remain an important question. A direct neurotoxic effect of P2X7 stimulation on RGCs is the simplest explanation and a likely contributor to RGC death. The P2X7 receptor has been located in RGCs [69,70], and expression has been shown in humans in the inner retina [13]. Furthermore, the direct stimulation of isolated RGCs causes Ca^2+^-mediated degeneration [12]. However, since organotypic retinal cultures retain both the regional morphology of the retina and cell-to-cell signalling, including synaptic connectivity and neuronal–glial interactions, an indirect mechanism of P2X7-mediated RGC death is also possible and, indeed, likely to be contributing within such a complex system. The potential mechanisms could include a P2X7-induced production of reactive oxygen species by microglia [71] or the release of other pro-inflammatory/neurotoxic factors, for example, tumour necrosis factor-alpha (TNFα) [17]. Müller cells and astrocytes, in addition to microglia, become activated in experimental models of glaucoma via mechanisms involving the P2X7 receptor [16,29], implying the existence of a complex interplay between the glial cells of the retina and neurodegenerative RGC death. Furthermore, both Müller cells and astrocytes have been shown to release ATP in response to physiological/pathophysiological stimuli [72,73,74], which provide a source of ATP for P2X7 activation.

Interestingly, the neuroinflammatory processes identified in the present study occur in the absence of added factors that prime immune cells to upregulate the machinery for the synthesis and release of IL-1β. It is commonplace for experiments investigating the role of P2X7 activation in IL-1β release to expose cells/tissue to the bacterial coat component lipopolysaccharide (LPS) to initiate and consequently upregulate the required intracellular machinery, including the NLRP3 inflammasome and pro-IL-1β. We have shown in the present study that, in the intact human retina, P2X7 stimulation leads to IL-1β release without exposure to bacterial components. This is important, since, within the retina and other parts of the CNS, the in vivo activation of microglia is very rarely a result of LPS and TLR-4 activation; many factors could be involved in increasing IL-1β expression in preparation for a neuroinflammatory response, but it is important to recognise the central role of ATP release and P2X7 activation.

We have presented evidence that extracellular ATP regulates IL-1β via the P2X7 receptor in the human retina. The ATP/P2X7/IL-1β axis has the capacity to critically influence the balance between neurodegenerative and neuroprotective mechanisms in the human retina. Demonstrating neuroinflammatory pathways in the human CNS contributes to the body of evidence placing these pathways centrally in the pathophysiological mechanisms of neurodegenerative diseases, including glaucoma.

## 4. Materials and Methods

### 4.1. Human Organotypic Retinal Cultures (HORCs)

Donor human eyes were obtained within 24 h post mortem from the East Anglian Eye Bank (EAEB) with ethical approval from the Norfolk Research Ethics Committee and under the tenets of the Declaration of Helsinki. Eyes with known ocular disease were excluded. Human organotypic retinal explant cultures (HORCs) were produced as described previously [46,47]. Briefly, the human retina was dissected, and 4 mm-diameter punch explants, taken from the paramacular region, were cultured RGC side upwards in serum-free Dulbecco’s Modified Eagle Medium (DMEM)/HamF12 in 35 mm cell culture dishes. All media were supplemented with gentamicin (50 µg/mL). HORCs were incubated at 35 °C in a humidified atmosphere of 95% air/5% CO_2_.

In experiments where 2′,3′-O-(4-benzoylbenzoyl)-ATP (BzATP; Sigma-Aldrich, Gillingham, UK) was used, it was incubated with HORCs in serum-free DMEM/HamF12 for up to 48 h. All antagonist (Brilliant Blue G; Sigma-Aldrich, Gillingham, UK) and human IL-1ra (PreproTech EC, Horsham, UK), where used, were incubated with HORCs in serum-free DMEM/HamF12 for one hour prior to addition of BzATP. In experiments involving recombinant human IL-1β (Sigma-Aldrich, Gillingham, UK), the IL-1β was added at the same time as BzATP.

### 4.2. Suspended Bead Array Analysis of Cytokines and Growth Factors

For analysis of the cytokines and growth factors secreted into culture medium by HORCs, the Bio-Plex suspended multiplex bead array assay kit (Bio-Rad, Watford, UK), using a 96-well plate format, was utilised according to manufacturer instructions. The plate was read on the Bio-Plex flow cytometry system X Map-100 (Luminex, Austin, TX, USA). The accompanying Bio-Plex Manager software (Bio-Rad, Watford, UK) calculated the concentration of each cytokine and growth factor in the sample medium using the standard curve derived from the recombinant cytokine and growth factor standards provided in the assay kit.

### 4.3. Enzyme-Linked Immunosorbant Assay (ELISA)

The human IL-1β enzyme-linked immunosorbant assay (ELISA) Ready-Set-Go kit (eBioscience, Horsham, UK) was used for quantitative measurements of IL-1β protein in the culture medium of HORCs. The 96-well ELISA plate (Corning Costar 9018) was coated with anti-human IL-1β capture antibody (clone CRM56). The detection antibody was a biotin-conjugated anti-human IL-1β detection antibody (clone CRM57); this antibody detects both pro- and mature IL-1β. Recombinant human IL-1β (Sigma-Aldrich, Gillingham, UK) was used to determine the standard curve.

### 4.4. Quantitative Real-Time Polymerase Chain Reaction (QRT-PCR)

Total RNA was extracted from HORCs using the RNeasy Mini Kit column-based method (Qiagen, Manchester, UK). A DNaseI incubation step was included to eliminate contamination from genomic DNA. Total RNA was measured using a NanoDrop ND-1000 spectrophotometer (NanoDrop Technologies, Wilmington, DE, USA) and reverse transcribed. The ABI Prism 7700 Sequence Detection System (Applied Biosystems, Horsham, UK) was used for QRT-PCR. The test probe/primer sets used were for Human *THY-1* (Hs00174816_m1, Applied Biosystems, Horsham, UK) and Human *IL-1β* (Hs00174097_m1, Applied Biosystems, Horsham, UK). Cytochrome c-1 (*CYC1*; HK-DD-hu-300; Primer design, Southampton, UK) and topoisomerase DNA I (*TOP1*; custom primers from Roche Diagnostics, Welwyn, UK with Roche Diagnostics Human Universal Probe Library Set, Welwyn, Roche) were used as housekeeping genes. Using TaqMan^®^ PCR Master Mix (Applied Biosystems, UK), PCR amplification was carried out at 50 °C for 2 min, 95 °C for 10 min, and then 40 cycles of 15 s at 95 °C and 1 min at 60 °C. The geometric means of C_T_ values of *CYC1* and *TOP1* were used to normalise the data for *THY-1* in the HORC experiments.

### 4.5. Immunohistochemistry

The HORCs were fixed in 4% paraformaldehyde for 24 h and then cryopreserved in 30% sucrose in phosphate-buffered saline (PBS) for a further 24 h. Subsequently, HORCs were placed into cryostat block moulds filled with optimal cutting temperature medium (Sakura Finetek, Alphen aan den Rijn, The Netherlands) and frozen on dry ice. Retinal slices (13 μm) were cut using a Hacker Bright OTF 5040 cryostat (Bright Instruments, Huntingdon, UK) and collected on 3′aminopropyl-triethoxyl silane (TESPA)-coated glass slides. Immunohistochemical labelling of NeuN in HORC sections has been described previously [46]. The primary monoclonal antibody for neuronal nuclei (NeuN) (Millipore, Gillingham, UK) was used at 1:200 and was incubated at 4 °C overnight. The secondary antibody was Alexa Fluor 488–conjugated (Invitrogen, Gillingham, UK) used at 1:1000, and incubation was for 2 h at room temperature. The primary polyclonal antibody for the extracellular domain of human IL-1R1 (GeneTex, Irvine, CA, USA) was used at 1:400 and was incubated at 4 °C overnight. The secondary antibody was Alexa Fluor 568-conjugated (Invitrogen, Horsham, UK) used at 1:1000, and incubation was for 2 h at room temperature. Samples were protected from light from this point onwards. Nuclei were counterstained with 4′,6-diamidino-2-phenyindole dilactate (DAPI) (1:100) for 10 min at room temperature prior to mounting in hydromount (National Diagnostics, Nottingham, UK) immunofluorescence mounting medium. A wide-field Zeiss Axiovert 200 M fluorescent microscope with a 100 W mercury arc lamp was used for immunofluorescent imaging. Zeiss Axiovision 4.7 software was used, and images were captured with a cooled monochrome CCD camera (Zeiss AxioCam, Cambourne, UK).

### 4.6. Image Analysis

The number of NeuN-labelled RGC layer cells was used as a measure of RGC death. Six non-consecutive immunohistochemical slices were prepared for each HORC, and the numbers of NeuN-stained RGC layer neurones were counted (in a masked fashion) in three 200 μm sections of each slice. The mean for the six slices was taken as one biological replicate. The extent of co-localization between IL-1R1 and NeuN was determined using the JACoP ImageJ plugin v1.54p [75].

### 4.7. Statistical Analysis

Significance was evaluated using SPSS software v17 using either Student’s *t*-test or one-way ANOVA followed by Tukey’s post hoc multiple comparison test. A *p*-value < 0.05 was considered statistically significant.

## Figures and Tables

**Table 1:** Protein concentrations of 27 cytokines and growth factors in medium from control HORCs and 100 μM BzATP-stimulated HORCs at 36 h. Measurements were conducted using suspended multiplex bead array assay. Data are presented as mean ± SEM; *n* = 4 donor eyes; * *p* < 0.05; Student’s *t*-test cf control.

Cytokine or Growth Factor	Mean (± S.E.M.) Protein Concentration in Control Medium (pg/mL)	Mean (± S.E.M.) Protein Concentration in 100 μM BzATP-Treated Medium (pg/mL)	Significance * *p* < 0.05
IL-2	0	0	
IL-1β	95.7 ± 20.0	295.3 ± 55.3	*
IL-1ra	663.7 ± 133.5	526.0 ± 28.7	
IL-4	26.6 ± 10.5	14.3 ± 5.2	
IL-5	0	0	
IL-6	5101.5 ± 965.3	6253.7 ± 1124.3	
IL-7	7.6 ± 3.9	6.6 ± 2.7	
IL-8	10,040.7 ± 1029.8	16,879.0 ± 10,321.1	
IL-9	213.4 ± 43.1	154.7 ± 13.7	
IL-10	33.9 ± 12.6	118.0 ± 17.0	*
IL-12	94.3 ± 10.1	126.7 ± 19.5	
IL-13	14.8 ± 4.9	43.2 ± 14.3	
IL-15	239.3 ± 52.5	196.2 ± 24.8	
IL-17	0	0	
TNF-α	738.7 ± 212.1	536.3 ± 60.0	
Interferon-γ (gamma)	1906.4 ± 465.0	1369.2 ± 131.6	
Interferon-Inducible Protein 10	56,210.7 ± 9465.3	31,524.1 ± 2788.1	
Granulocyte-Colony Stimulating Factor	2460.8 ± 682.6	3650.4 ± 424.9	
Granulocyte Monocyte-Colony Stimulating Factor	76.3 ± 54.8	0	
Monocyte Chemoattractant Protein-1 (Monocyte Chemoattractant and Activating Factor)	4697.6 ± 628.1	4093.5 ± 444.3	
Macrophage Inflammatory Protein-1alpha	1174.9 ± 96.9	1212.3 ± 54.1	
Macrophage Inflammatory Protein-1beta	6353.2 ± 456.7	5588.0 ± 201.1	
Eotaxin	448.3 ± 93.0	319.6 ± 28.1	
Regulated on Activation, Normal T Expressed and Secreted (RANTES)	390.9 ± 97.8	161.5 ± 21.4	
Vascular Endothelial Growth Factor	149.8 ± 55.5	629.4 ± 329.1	
Platelet Derived Growth Factor-BB	95.0 ± 59.6	83.9 ± 48.7	
Fibroblast Growth Factor-Basic	55.7 ± 24.7	39.0 ± 17.9	

## Data Availability

The data that support the findings of this study are available upon request from the corresponding author.
